# Phytochemical Composition and Cytoprotective Properties of the Endemic *Sideritis sipylea* Boiss Greek Species: A Valorization Study

**DOI:** 10.3390/ph15080987

**Published:** 2022-08-11

**Authors:** Silvia Di Giacomo, Antonella Di Sotto, Apostolis Angelis, Ester Percaccio, Annabella Vitalone, Marco Gullì, Alberto Macone, Evangelos Axiotis, Alexios Leandros Skaltsounis

**Affiliations:** 1 Department of Physiology and Pharmacology “V. Erspamer”, Sapienza University of Rome, P.le Aldo Moro 5, 00185 Rome, Italy; 2 Department of Pharmacognosy and Natural Products Chemistry, Faculty of Pharmacy, National and Kapodistrian University of Athens, 15771 Athens, Greece; 3 Department of Biochemical Science “A. Rossi Fanelli”, Sapienza University of Rome, P.le Aldo Moro 5, 00185 Rome, Italy; 4 Natural Products Research Center “NatPro Aegean”, Gera, 81106 Lesvos, Greece

**Keywords:** Greek medicinal plants, UPLC-HRMS/MS, North Aegean, mountain tea, phenolics, antioxidant activity, chelating activity, advanced glycation end products, oxidative stress, antiproliferative activity

## Abstract

*Sideritis sipylea* Boiss. (Fam. Lamiaceae) is an endemic plant of the North Aegean Islands (Greece), commonly known as ironwort. Traditionally, its aerial parts have been used to relieve several ailments, especially gastrointestinal disorders, however, with scant knowledge about the pharmacological basis. In the present study, an endemic *S. sipylea* Greek species from Lesvos Island has been characterized for phytochemical composition and biological activities, in order to give a possible scientific basis to its traditional use and to highlight a further nutraceutical interest as a source of bioactive phytochemicals and extracts. Three different fractions obtained from a methanolic extract of *S. sipylea* aerial parts by using ethyl acetate with 10 (S10), 20 (S20), and 50% (S50) methanol as fractionation solvents were phytochemically characterized. Moreover, their antioxidant power and cytoprotective activity in different human cell lines were evaluated. The phytochemical analysis highlighted the presence of flavonoids, iridoids, and phenolic acids in all the tested samples. Particularly, the S10 fraction mainly contained iridoids, while S20 and S50 lavandulifolioside and chlorogenic acid, respectively. The fractions also showed antioxidant properties, S10 and S20 being the most potent. When assessed in human cholangiocytes, they counteracted the cytotoxicity of the tBOOH pro-oxidant agent, by reducing ROS levels and affecting GSH antioxidant system. The present findings highlight a possible interest in S10 and S20 fractions from *S. sipylea* as sources of bioactive molecules and stimulate further studies in order to characterize their possible application for nutraceutical and pharmaceutical purposes.

## 1. Introduction

Ironwort or *Sideritis* spp. includes about 150 species and subspecies, found mainly in the Mediterranean countries, the Canary Islands, and Western Asia [1]. They grow in temperate regions and tropical climates in the northern hemisphere, from the Bahamas to western China and from Germany to Morocco [2,3]. In the Greek territory, the genus comprises eight species and seven subspecies [4].

Preparations of various species of the genus *Sideritis* are used in folk medicine against several diseases, as it has been widely reported in many ethnopharmacological studies [5,6,7,8]. Decoctions and infusions, derived from the aerial parts of the plant, are used traditionally for the treatment of many ailments, especially as gastroprotective, antiulcerative [9], or analgesic [10]. Moreover, the antimicrobial activity against Gram-positive or Gram-negative of its essential oils or the antifungal activity against *Candida albicans* of *Sideritis* extracts has been previously reported [11,12,13]. The most popular Greek species, *S. scardica* Griseb., known as Greek mountain tea, has been widely used in traditional medicinal preparations against stomach complaints and as a tonic, diuretic, and detoxification agent [14]. Decoctions of its aerial parts have been typically employed for infections of the upper respiratory system (common cold) [3]. It has recently been observed that extracts of *S. euboea* and *S. scardica* species alone or in combination possessed anti-Alzheimer’s activity, improving memory in experimental animals and reducing total *β*-amyloid deposition [15]. Additionally, in-vivo experiments in mice showed positive effects of *Sideritis scardica* extracts in mental disorders associated with dysfunctional monoaminergic neurotransmissions, such as anxiety disorders, major depression, attention deficit hyperactivity disorder (ADHD), and hyperactivity [15,16]. It is of utmost importance that on 2016 the Committee on Herbal Medicinal Products (HMPC) of the European Medicines Agency (EMA) published an assessment report for the establishment of a European Union herbal monograph on the traditional use of *S. scardica*, *S. clandestina* (Bory and Chaub.) Hayek, *S. raeseri* Boiss., and *S. syriaca* L. for the relief of cough associated with cold as well as for the relief of mild gastrointestinal discomfort [17].

Chemical constituents such as terpenoids, flavonoids, sterols, iridoids, lignans, and coumarins have been identified in *Sideritis* spp., with many of the secondary metabolites thereof being known for their biological activity [5,18,19]. Decoctions and polar extracts of the genus *Sideritis* have been reported to possess antioxidant properties, due to their high content of flavonoid aglycones [19]; moreover, anti-inflammatory, antibacterial, cytotoxic, and anti-tumor effects, likely ascribed to the presence of kaurane diterpenes (e.g., foliol, sidol, linearol) [20] and other phenolic constituents, particularly phenylpropanoid glycosides (i.e., leucoseptoside, verbascoside, and martynoside) [18].

*Sideritis sipylea* Boiss. is an endemic plant of the Balkans distributed mainly across the Greek islands of the North Aegean Region (i.e., Lesvos, Chios, Samos, and Ikaria), as well as in the coastal zone of Asia Minor. The botanical characteristics of this species have been extensively referred to previously [1,21]. Surveys reported its ethnopharmacological uses mainly in the coastal zone of Asia Minor [22,23], with a single report referring to its use in Greek islands [5] as decoctions to relieve common cold and gastrointestinal disorders. Despite the traditional interest, very few studies have been performed to reveal the bioactivities and mechanisms of its extracts and to clarify the bioactive compounds. The antimicrobial activity of the essential oil [12] or isolated diterpenes [24] has been reported. Moreover, in 2006 the antioxidant capacity of different polarity extracts of *S. sipylea* has been studied showing promising results but without a specific correlation with their phytochemical composition [25]. Conversely, Tomu et al. [26] isolated and identified the polar constituents of both methanolic extract and infusion of *S. sipylea* from Samos island, highlighting anti-acetylcholinesterase, and cytotoxic activities.

In a previous study, the total phytochemical profile and radical scavenging activity of six different extracts from the aerial parts of *S. sipylea* have been characterized and the methanolic extract was found to possess the strongest activity, thus increasing our interest in its characterization [22].

In line with this evidence, in the present study, chromatographic separation of the *S. sipylea* methanolic extract [22] has been carried out, in order to better concentrate the bioactive constituents and to deeply evaluate its pharmacological profile, especially focusing on the antioxidant and cytoprotective properties. Owing to the known involvement of oxidative stress in a wide range of ailments including gastrointestinal, respiratory, and immune system ones [27], maintaining or restoring the redox state by using natural products could be an interesting strategy to prevent disease development and avoid their exacerbation. To perform the study, the secondary metabolite composition of the samples was evaluated by spectrophotometric and chromatographic analysis. Moreover, a battery of in vitro assays was applied to unravel both direct and indirect potential antioxidant mechanisms. The ability of the fractions to counteract the oxidative stress induced by the pro-oxidant agent tBOOH, leading to cytoprotective effects, has been evaluated in human cell models.

## 2. Results

### 2.1. Phytochemical Analysis

An enriched methanol extract of *S. sipylea* Boiss aerial parts was fractionated by VLC as described in detail in Section 4.2. From all the different fractions that emerged and based on both their final weights (Table S1, Supplementary Material) and overall qualitative profile after UPLC-ESI (-)-HRMS (Supplementary Material, Figure S2), we decided that the most promising extracts for further phytochemical analysis as well as for the in vitro experiments were the fractions EtOAc/MeOH (10%) (S10), EtOAc/MeOH (20%) (S20), and EtOAc/MeOH (50%) (S50).

#### 2.1.1. Spectrophotometric Analysis

High amounts of total polyphenols, tannins, and flavonoids were found in all the fractions (Table 1). Particularly, S10 and S20 samples contained the highest content of total polyphenols, while a 1.2-fold lower level was highlighted in S50 (Table 1). Regarding flavonoids, the S10 sample was the most enriched, their content being 2.4- and 3-fold lower in S20 and S50, respectively. Conversely, the tannin amounts among the fractions was almost similar (Table 1).

#### 2.1.2. Ultra-Performance Liquid Chromatography Coupled with High-Resolution Mass Spectrometry (UHPLC-HRMS) of S10, S20, and S50 Fractions

Based on detailed UPLC-HRMS/MS analysis of three different fractions (S10, S20, and S50, respectively), obtained from methanol total extract of wild *S. sipylea*, and by meticulous comparison of fragmentation patterns with literature data, 19 different secondary metabolites were identified (Table 2), belonging mainly to flavonoids, iridoids, and phenolic acids. The chemical structures of the identified compounds are presented in Supplementary Material, Figure S1.

More specifically, phenolic acids such as quinic acid and chlorogenic acid (5) were identified in the S50 fraction, while feruloylquinic acid (3) was only in S10 (Table 2). Furthermore, iridoids such as melittoside derivatives (1, 4) are identified in all three different fractions. Phenylethanoid glycosides such as lavandulifolioside (8), isoverbascoside (9), and leucoseptoside A (10) are identified in all three different fractions, while echinacoside (7) only in S20 and S50. The different flavonoids are flavones and their glycosylated forms such as isoscutelarein 7-*O*-allosyl-(1→2)-glucoside (12), apigenin 7-*O*-glucoside (11), luteolin 7-*O*-allosyl-(1→2)-[6″-*O*-acetyl]-glucoside (13), apigenin (17), 4′-*O*-methylisoscutellarein 7-*O*-allosyl-(1→2)-glucoside (14) and 4′-*O*-methylisoscutellarein 7-*O*-allosyl-(1→2) [6″-*O*-acetyl]-glucoside (15), and finally 4′-*O*-methylisoscutellarein 7-*O*-[6″′-*O*-acetyl]-allosyl-(1→2)-[6″-*O*-acetyl]-glucoside (18) (Table 2).

### 2.2. Radical Scavenger Activity

**Figure 1:**
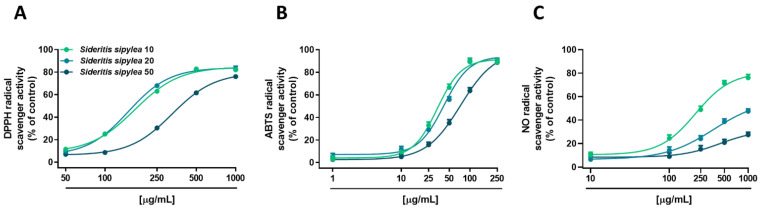
Scavenger activity towards (**A**) DPPH, (**B**) ABTS, and (**C**) NO radicals of S10, S20, and S50 fractions from *Sideritis sipylea* Boiss. Data represent the average and standard error of at least three independent experiments (*n* = 3).

Under our experimental conditions, all the fractions from *S. sipylea* showed radical scavenger activity, although with different potency and efficacy (Figure 1 and Table 3). Particularly, both S10 and S20 samples inhibited DPPH radical starting from 100 µg/mL, achieving the maximum effect of about 83% at the concentration of 500 µg/mL (Figure 1A), while the inhibition by S50 appeared at 250 µg/mL and became complete (about 76%) at 1000 µg/mL (Figure 1A). This different behavior was also confirmed by the IC_50_ value of S50 which was doubled with respect to those of S10 and S20 fractions (Table 3).

When assayed against an ABTS radical, all the samples were able to fully scavenge it, both S10 and S20 fractions being the most potent samples, with a maximum inhibition at the concentration of 100 µg/mL (Figure 1B). Conversely, S50 reached the maximum effect by about 89% at 250 µg/mL (Figure 1B). The IC_50_ values corroborated these behaviors, that of S50 being almost doubled with respect to those of the other samples (Table 3).

At last, in the nitric oxide assay, S10 produced a strong scavenging activity (about 76% radical inhibition) at the highest tested concentration of 1000 μg/mL (Figure 1C). Instead, S20 and S50 fractions showed moderate (lower than 50%) and weak (lower than 20%) activity, respectively (Figure 1C). The IC_50_ value of the S10 fraction was equal to 204.4 (CL 108.5–396.6), while those of the other samples were not evaluable, as the maximum effect was lower than 80% (Table 3). As expected, in all the assays, the positive control trolox exhibited a more potent scavenging effect, as shown by the IC_50_ values reported in Table 3.

### 2.3. Iron Chelating and Reducing Activity

The ability of the samples to chelate or reduce iron ions, which represents a mechanism of indirect antioxidant activity, was investigated too.

**Figure 2:**
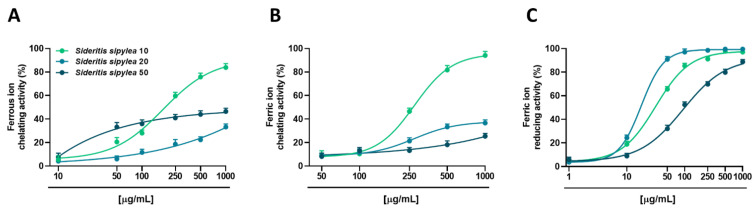
Ability of S10, S20, and S50 fractions from *Sideritis sipylea* Boiss to chelate ferrous (**A**) and ferric (**B**) ions, and to reduce ferric (**C**) ones. Data represent the average and standard error of at least three independent experiments (*n* = 3).

Under our experimental conditions, all the samples were able to chelate both ferrous and ferric ions, with the S10 fraction having great potency and efficacy (Figure 2A). Indeed, at the highest concentration tested (1000 µg/mL), S10 determined a maximum chelating effect of about 84% and 94% towards ferrous and ferric ions, while S20 and S50 fractions showed only a moderate (lower than 50%) activity. The IC_50_ value of the S10 fraction was equal to 188.9 (CL 147.5–280.1) µg/mL and 273 (CL 255.5–294.0) µg/mL, respectively (Table 3).

All the fractions were also able to reduce ferric ions, although the highest potency and efficacy was from S20. Indeed, it determined a 90% reducing effect already at the concentration of 50 µg/mL, reaching the maximum activity at 500 µg/mL. Conversely, a 90% reducing effect was achieved by S10 and S50 fractions at concentrations 5- and 20-fold higher than S20. This behavior was confirmed by the IC_50_ values, which were about 2- and 5-fold higher in S10 and S50 samples with respect to the S20 one (Table 3). As expected, the positive controls trolox and quercetin exhibited a more potent chelating and reducing effect, as displayed by the IC_50_ values (Table 3).

### 2.4. Inhibition of Advanced Glycation End-Product (AGE) Formation

The ability of *S. sipylea* fractions to inhibit AGE formation was also evaluated as a mechanism of indirect antioxidant activity. Indeed, these products are produced under different stress conditions and are involved in the development and progression of different pathologies, due to their pro-oxidant effect [28].

Under our experimental conditions, the samples were able to block AGE generation, although with a moderate effect (Figure S3). Particularly, S20 was the most effective reaching a maximum effect of about 50% at the highest concentrations (250 µg/mL), followed by S10 and S50 samples (45% and 38% AGE formation inhibition, respectively). The IC_50_ values of the samples were not evaluable, while a strong effect was found for the positive control rutin, as shown by the IC_50_ value (Table 3).

### 2.5. Cytotoxicity of S10, S20, and S50 Fractions in Cell Lines

Being the *Sideritis* spp. extracts traditionally used to enforce the immune system and to relieve several ailments, among which respiratory and gastrointestinal ones [11,22,29], the cytotoxicity of the tested fractions was evaluated in nonmalignant RAW264.7 (mouse macrophages), BEAS-2B (human bronchial epithelium cells), and H69 (human intrahepatic cholangiocytes) cell lines. Both MTT and Neutral Red assays were performed to assess the sample cytotoxicity due to possible damage at the mitochondrial or lysosomal level. These studies allowed not only to evaluate the tolerability of the samples but also to select the nontoxic concentrations to be further tested for cytoprotective properties.

**Figure 3:**
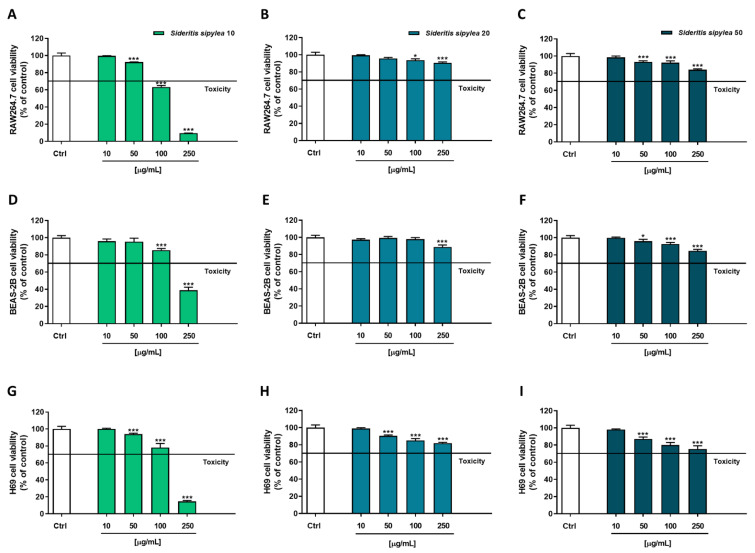
Cytotoxicity of S10, S20, and S50 fractions from *Sideritis sipylea* Boiss in nonmalignant mouse macrophages RAW264.7 (**A**–**C**), human bronchial epithelium BEAS-2B cells (**D**–**F**), and human intrahepatic cholangiocytes H69 (**G**–**I**) determined by MTT assay after 24 h of exposure. Data displayed as mean ± SE of at least three independent experiments (*n* = 3). * *p* < 0.05 and *** *p* < 0.001 vs. control (one-way ANOVA followed by Dunnett’s multiple comparison post-test).

The MTT assay highlighted that S10 was the most cytotoxic fraction, especially at the highest tested concentration (250 µg/mL), which induced a reduction of cell viability by about 90%, 61%, and 85% in RAW264.7 (Figure 3A), BEAS-2B (Figure 3D), and H69 (Figure 3G), respectively. However, early significant signs of toxicity were already present at the concentration of 50 µg/mL in RAW264.7 and H69 (8% and 6% inhibition of cell viability) cells, and at 100 µg/mL in BEAS-2B (15% inhibition of cell viability). Conversely, S20 and S50 fractions did not affect cell viability. Indeed, although for both samples a significant inhibitory effect was detected starting from the 50 µg/mL concentration, in almost all the cell lines, the cutoff value of 70% with respect to the control was never reached, so the treatments could not be considered as cytotoxic.

**Figure 4:**
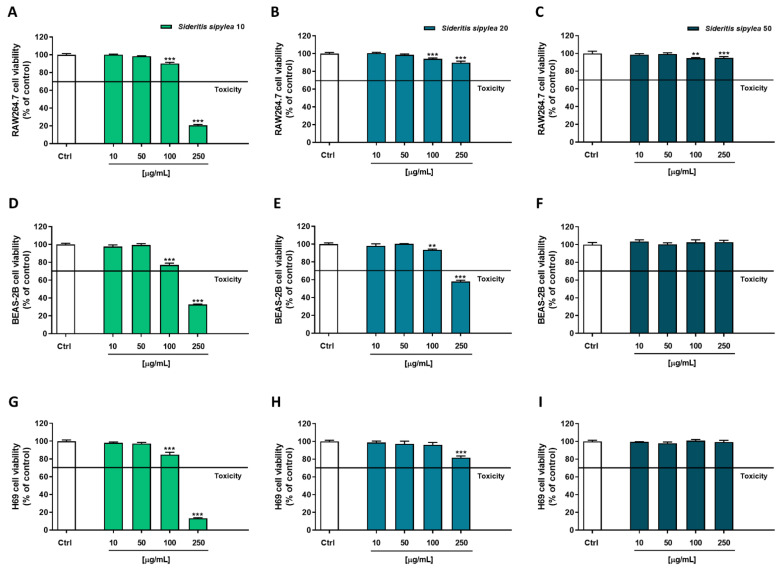
Cytotoxicity of S10, S20, and S50 fractions from *Sideritis sipylea* Boiss in nonmalignant mouse macrophages RAW264.7 (**A**–**C**), human bronchial epithelium BEAS-2B cells (**D**–**F**), and human intrahepatic cholangiocytes H69 (**G**–**I**) determined by Neutral Red assay after 24 h exposure. Data displayed as mean ± SE of at least three technical replicates from two experiments (*n* = 3). ** *p* < 0.01 and *** *p* < 0.001 vs. control (one-way ANOVA followed by Dunnett’s multiple comparison post-test).

In the Neutral Red assay, a similar behavior was observed (Figure 4): once again, S10 was the most cytotoxic fraction, determining early signs of toxicity starting from the concentration of 100 µg/mL in all the cell lines. Indeed, a reduction of cell viability by about 10%, 23%, and 15% was highlighted in RAW264.7, BEAS-2B, and H69 cells, respectively. As expected, the most cytotoxic effect was achieved at the highest tested concentration of 250 µg/mL, the reduction of cell viability being equal to 79%, 67%, and 87% in RAW264.7, BEAS-2B, and H69 cells, respectively.

Regarding the S20 sample, BEAS-2B cells were the most affected: a 6% and 42% reduction of viability was observed at 100 µg/mL and 250 µg/mL, respectively; conversely, a slight inhibition of cell viability was highlighted in RAW264.7 and H69 cells, a maximum 10% to 18% effect being achieved at the highest tested concentration. At last, the S50 sample did not show any cytotoxicity in BEAS-2B and H69 cells, with a lower than 10% inhibition of cell viability in RAW264.7 cells.

Based on the present results, the following ranges of nontoxic concentrations were chosen to be tested in the subsequent cytoprotecting studies: 10–100 µg/mL for S10 and 10–250 µg/mL for both S20 and S50 fractions.

### 2.6. Cytoprotective Activity of S10, S20, and S50 Fractions towards the Oxidative Damage Induced by tBOOH

To evaluate the antioxidant activity of *S. sipylea* fractions in biological systems, the pro-oxidant agent tBOOH has been exploited. Indeed, this compound is metabolized by cytochrome P450 to free radical intermediates which can subsequently lead to oxidative damage, at both the mitochondria and lysosomal level [30,31]. Therefore, MTT and Neutral Red assays were carried out in order to highlight the potential cytoprotection of *S. sipylea* fractions towards tBOOH damage. Particularly, a 24 h pretreatment protocol with the samples was applied followed by 3 h of exposure to the oxidant agent.

**Figure 5:**
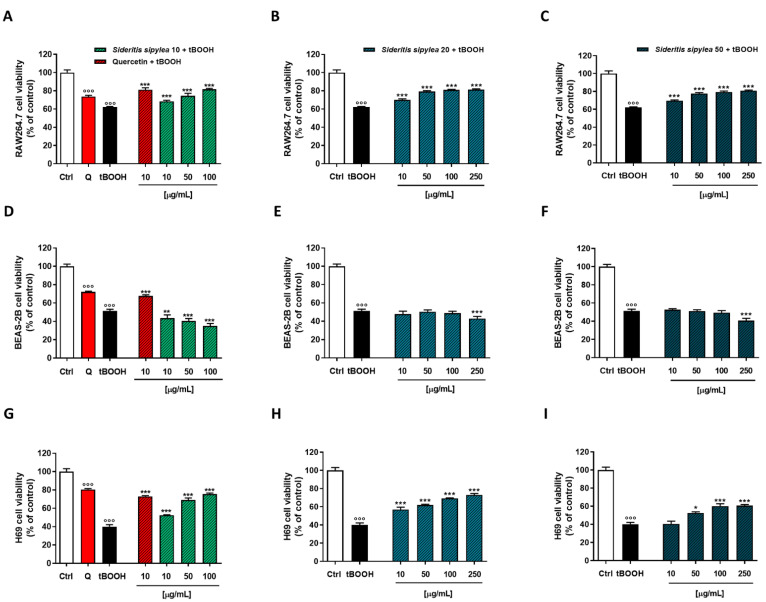
Cytoprotective activity of the positive control quercetin (Q) and of S10, S20, and S50 fractions from *Sideritis sipylea* Boiss towards the tBOOH-induced oxidative damage in nonmalignant mouse macrophages RAW264.7 (**A**–**C**), human bronchial epithelium BEAS-2B cells (**D**–**F**), and human intrahepatic cholangiocytes H69 (**G**–**I**) determined by MTT assay. Data displayed as mean ± SE of at least three technical replicates from two experiments (*n* = 3). °°° *p* < 0.001 vs. control (Student’s *t*-test); * *p* < 0.05, ** *p* < 0.01, and *** *p* < 0.001 vs. tBOOH (one-way ANOVA followed by Dunnett’s multiple comparison post-test).

The MTT assay highlighted that our cellular models had a different sensitivity to the damage from tBOOH: H69 normal cholangiocytes were the most sensitive with a reduction of cell viability by about 60%, while inhibition of cell viability by about 38% and 49% was observed in RAW264.7 and BEAS-2B, respectively. As expected, the positive control quercetin at the nontoxic concentration of 10 µg/mL was able to counteract the oxidative damage of tBOOH; indeed, the cell viability increased by about 19%, 16%, and 33% in RAW264.7, BEAS-2B, and H69 cells, respectively (Figure 5).

Regarding *S. sipylea* fractions, all the samples exerted protective effects, S10 and S20 being the most potent and effective. Particularly, in RAW264.7, S10 was able to counteract tBOOH damage by increasing cell viability starting from the lowest concentration of 10 µg/mL (+6%): the maximum 20% effect was achieved at 100 µg/mL. Additionally, S20 and S50 samples showed cytoprotective activities although lower than S10: they determined a maximum 19% cytoprotection at the concentration of 250 µg/mL, despite a slightly higher effect than S10 at the lowest concentration (Figure 5A–C).

In H69 cells, S20 was the most potent sample. It determined a 17% increase of cell viability with respect to tBOOH at the lowest tested concentration, while S10 had a 12% one; no effect was observed for S50. Conversely, S10 was the most effective among *S. sipylea* fractions, inducing a rising of cell viability by about 36% at the concentration of 100 µg/mL against the 29% and 20% induced by S20 and S50, respectively (Figure 5D–F).

Unexpectedly, none of the samples were able to counteract the oxidative damage of tBOOH in BEAS-2B cells; even better, a reduction of cell viability by about 16% for S10, 8% for S20, and 10% for S50 were highlighted at the highest tested concentration (Figure 5G–I).

**Figure 6:**
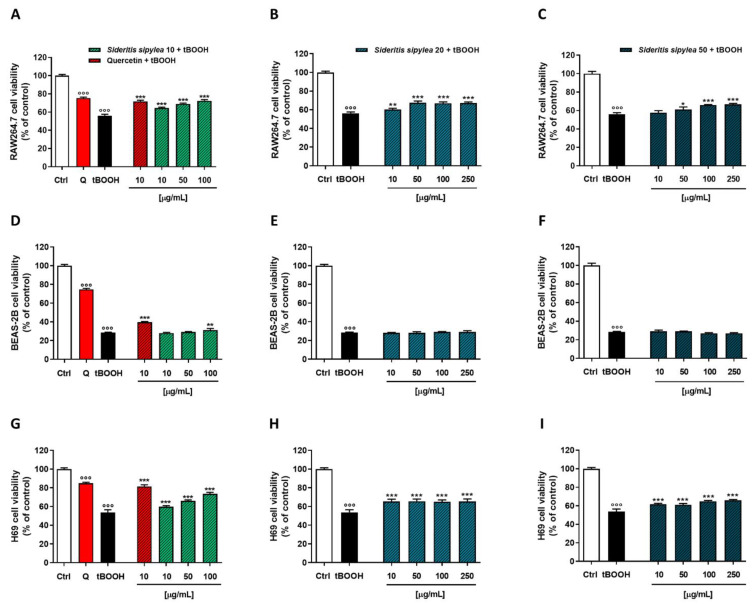
Cytoprotective activity of the positive control quercetin (Q) and S10, S20, and S50 fractions from *Sideritis sipylea* Boiss towards the tBOOH-induced oxidative damage in nonmalignant mouse macrophages RAW264.7 (**A**–**C**), human bronchial epithelium BEAS-2B cells (**D**–**F**), and human intrahepatic cholangiocytes H69 (**G**–**I**) determined by Neutral Red assay. Data displayed as mean ± SE of at least three technical replicates from two experiments (*n* = 3). °°° *p* < 0.001 vs. control (Student’s *t*-test); * *p* < 0.05, ** *p* < 0.01, and *** *p* < 0.001 vs. tBOOH (one-way ANOVA followed by Dunnett’s multiple comparison post-test).

In the Neutral Red assay, BEAS-2B cells were the most sensitive to the oxidative damage of tBOOH; indeed, it determined a 72% reduction in cell viability. Conversely, a similar effect was observed in RAW264.7 and H69 cells, namely an almost 45% inhibition of cell viability with respect to the control (Figure 6). The pretreatment with quercetin protected the cells from tBOOH damage, as highlighted by the increase of the cell viability by about 16%, 11%, and 28% in RAW264.7, BEAS-2B, and H69 cells, respectively (Figure 6).

Similar to the MTT assay, all *S. sipylea* fractions were able to counteract the tBOOH oxidative damage. Particularly, in RAW264.7 cells, the S10 sample was the most potent and effective: it determined early cytoprotection signs already at the lowest concentration (+8% cell viability), reaching the maximum effect of 16% at the highest one. Additionally, S20 and S50 samples were able to reduce the cytotoxicity induced by tBOOH (+11% cell viability at the highest tested concentration) but to a lesser extent (Figure 6A–C).

In H69 normal cholangiocytes, greater cytoprotection was observed for the S10 sample. Indeed, at 100 µg/mL, an increase of cell viability by about 20% with respect to tBOOH was found. Instead, a similar behavior as in RAW264.7 cells was observed for S20 and S50 fractions: a maximum 12% cytoprotective effect was achieved only at the highest concentration of 250 µg/mL (Figure 6D–F).

At last, none of the samples was able to counteract the oxidative damage of tBOOH in BEAS-2B cells, except for S10 which determines a slight but significant increase in cell viability with respect to the pro-oxidant agent (Figure 6G–I).

Based on these results and considering the high cytoprotection shown by our samples in H69 cells, this cell line was selected to further investigate the mechanisms by which *S. sipylea* fractions exerted their protective effects against tBOOH damage.

### 2.7. Sideritis sipylea Fractions Counteract tBOOH-Induced Oxidative Stress by Increasing GSH Levels

To evaluate the ability of our samples to counteract oxidative stress induced by tBOOH in H69 cells, the levels of ROS were measured through the DCFH-DA assay. Particularly, a protocol consisting of a 24 h pretreatment with the samples followed by a 3 h exposure with tBOOH was applied.

**Figure 7:**
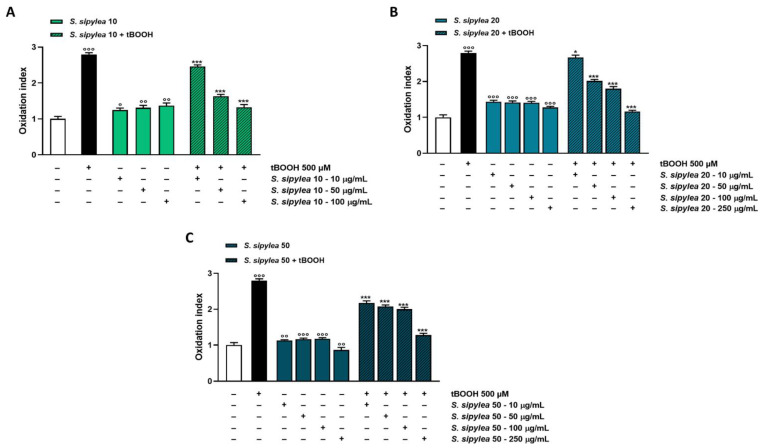
Effect of S10 (**A**), S20 (**B**), and S50 (**C**) fractions from *Sideritis sipylea* Boiss on the ROS levels induced by the pro-oxidant agent tBOOH in H69 cells. ROS levels are expressed as oxidation index with respect to the basal levels. Data are mean ± SE from at least three independent experiments (*n* = 3). ° *p* < 0.05, °° *p* < 0.01, and °°° *p* < 0.001 vs. control (one-way ANOVA followed by Dunnett’s multiple comparison post-test); * *p* < 0.05 and *** *p* < 0.001 vs. tBOOH (one-way ANOVA followed by Dunnett’s multiple comparison post-test).

**Figure 8:**
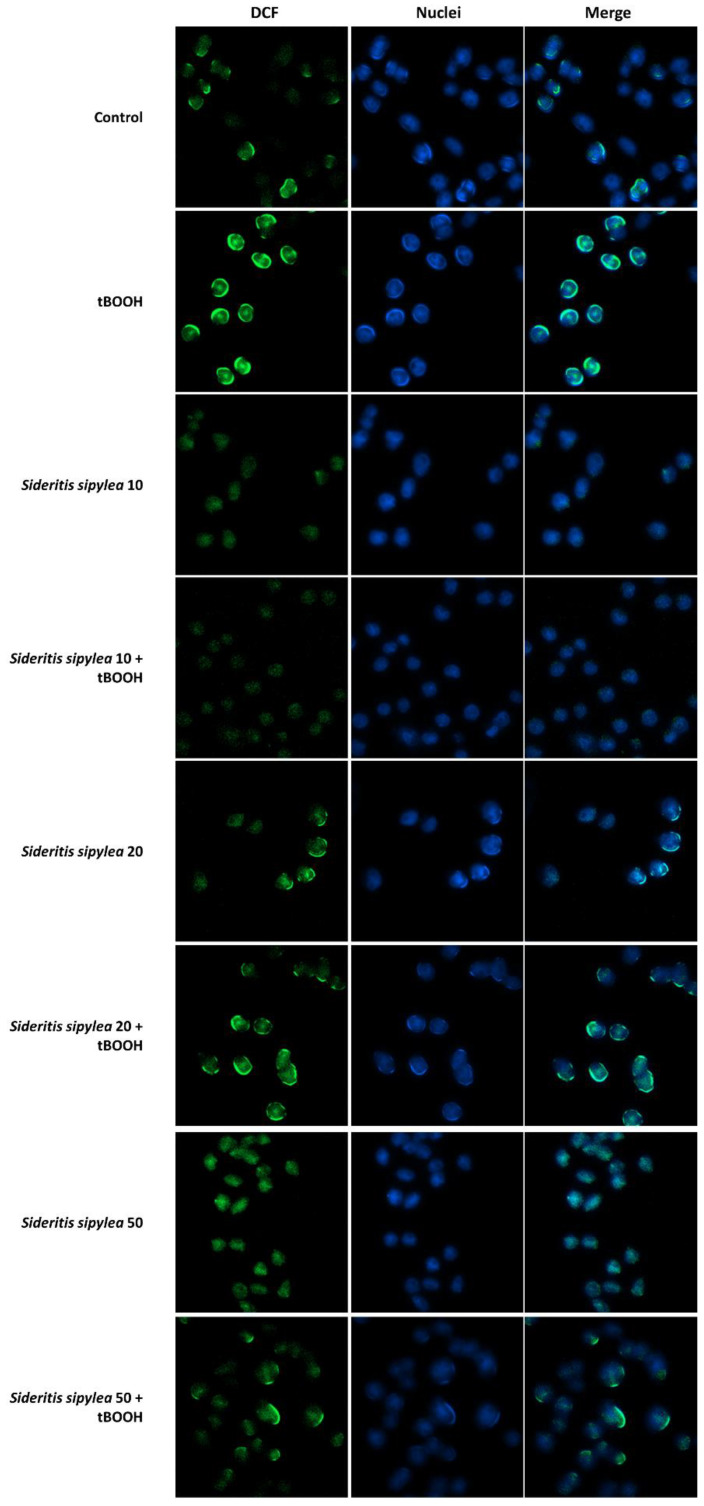
Representative images of intracellular ROS after treatment of H69 normal cholangiocytes with tBOOH (500 µM) and S10, S20, and S50 fractions (100 µg/mL) alone or in combination with the pro-oxidant agent. Original magnification 10X.

Under our experimental conditions, the oxidative agent induced an increase in ROS production by about 3-fold with respect to the control (Figure 7 and Figure 8).

Additionally, *S. sipylea* fractions determined a rise of ROS basal level, but to a lesser extent (from about 1.1-fold to a maximum of 1.4-fold) than tBOOH, within the tested concentrations. Only S50 at the highest tested concentration produced a slight but significant lowering of ROS by about 1.1-fold (Figure 7 and Figure 8).

Conversely, in the cytoprotective studies, all the samples were able to hinder ROS generation induced by tBOOH, S10 being the most active. Particularly, it lowered the ROS production by about 1.7-fold at the concentration of 50 µg/mL and by 2.2-fold at 100 µg/mL, thus bringing oxidative stress almost to baseline (Figure 7A and Figure 8). Additionally, S20 and S50 were effective in counteracting tBOOH-induced ROS formation, although they acted at higher concentrations. Indeed, a reduction of ROS level by about 2.3- and 2.2-fold, respectively, was achieved at 250 µg/mL, thus at a concentration 2.5-fold higher than that of S10 (Figure 7B,C and Figure 8).

**Figure 9:**
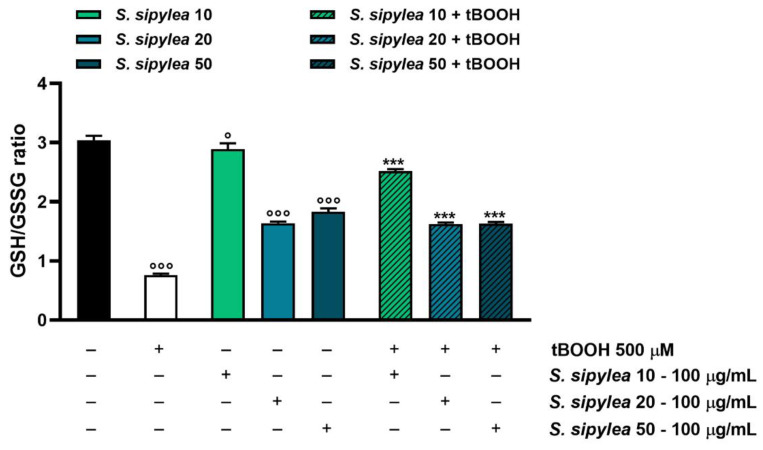
Effect of S10, S20, and S50 fractions from *Sideritis sipylea* Boiss on the GSH/GSSG ratio after treatment with the pro-oxidant agent tBOOH in H69 cells. GSH and GSSH were evaluated in cell lysates and calculated with respect to the calibration curves of GSH and GSSG. Data are mean ± SE from at least three independent experiments (*n* = 3). ° *p* < 0.05 and °°° *p* < 0.001 vs. control (one-way ANOVA followed by Dunnett’s multiple comparison post-test); *** *p* < 0.001 vs. tBOOH (one-way ANOVA followed by Dunnett’s multiple comparison post-test).

Considering that tBOOH detoxification takes place through conjugation with reduced glutathione (GSH) so generating oxidized glutathione (GSSG), the possible modulation of this target by *S. sipylea* fractions was investigated too. As expected, the pro-oxidant agent highly reduced the GSH/GSSG ratio by about 4-fold, meaning that GSH was depleted in favor of GSSG generation (Figure 9).

Unexpectedly, S20 and S50 fractions also highly reduced the GSH/GSSG ratio by about 1.9- and 1.7-folds, respectively. Particularly, both samples induced a reduction of GSH levels without affecting GSSG ones with respect to the control. Conversely, S10 determined a slight but significant decrease in the GSH/GSSG ratio; however, in this case, an increase in both GSH and GSSG levels was observed (Figure 9 and Table 4).

Despite that, the samples were able to hinder the GSH depletion induced by tBOOH: S20 and S50 samples increased the GSH/GSSG ratio by about 2-fold with respect to the pro-oxidant agent, by raising both GSH and GSSG concentrations (Figure 9 and Table 4). S10 was the most effective fraction since it induced a 3.3-fold increase in the GSH/GSSG ratio, almost restoring the basal cell levels (Figure 9). As shown in Table 4, the GSH and GSSG levels were significantly increased by S10 with respect to the control.

Overall, the present data are in agreement with the results obtained in ROS-level modulation and allow us to hypothesize an involvement of the GSH antioxidant system in the protective effects of *S. sipylea* fractions, especially for the S10 sample.

## 3. Discussion

Medicinal plants have been used since ancient times to relieve several ailments and to support general well-being. In many countries, such as Greece, they still maintain an important role in the therapy, especially for populations growing in the wild [22]. Despite that, only a few studies have been carried out to establish the scientific basis of their ethnopharmacological use. This represents an important field of research to support the traditional use of medicinal plants by highlighting the pharmacological mechanisms and the specific phytochemical composition needed to achieve the desired effect. Moreover, ethnopharmacological studies also allow to valorize medicinal plant biodiversity and to exploit them in pharmaceutical and nutraceutical fields. At last, ethnopharmacology represents an important tool for the sustainable use of medicinal plants to avoid an uncontrolled collection that can endanger botanical species [32,33].

*Sideritis* species are widely used in traditional Greek medicine, due to their several therapeutic properties ascribable to the presence of different compounds, among which phenolic acids, phenylpropanoid glycosides, flavonoids, and kaurene diterpenes [6]. Particularly, *S. scardica* is the most studied variety and is traditionally exploited to aid digestion, strengthen the immune system, and treat respiratory ailments [5]. However, other endemic *Sideritis* species are used for primary care by local people, which have limited access to conventional medicines [32]. Noteworthy, some studies have shown that plants belonging to the genus *Sideritis* (e.g., *S. scardica* and *S. raeseri*) and cultivated in the Balkans area contain similar phytochemicals, probably due to the peculiar pedoclimatic conditions; therefore, the same health benefits could be expected [34]. For example, *Sideritis sipylea* Boiss. is traditionally exploited for the same uses as *S. scardica*, although less characterized, especially from a pharmacological point of view.

In line with this evidence and on the basis of previous studies highlighting promising radical scavenging properties of a methanolic extract from the aerial parts of a *Sideritis sipylea* species, collected at Olympus Mount (Lesvos, Greece) [22], in the present study, we evaluated the phytochemical composition and biological activities of three different fractions from this extract, in order to better define the bioactive compounds and to better highlight a future interest in this species in the nutraceutical field.

The phytochemical analysis highlighted a peculiar composition of the fractions, characterized by a high amount of total polyphenols and flavonoids. Particularly, S10 was the most enriched and mainly contained isoverbascoside and flavone glycosides, such as 4′-*O*-methylisoscutellarein 7-*O*-allosyl-(1→2) [6′′-*O*-acetyl]-glucoside, which was also the most abundant in the original extract [22]. Apart from flavone glycosides, S20 was also characterized by the presence of echinacoside and lavandulifolioside, while S50 by quinic acid and chlorogenic acid. Additionally, iridoid derivatives were found in all the fractions.

To the best of our knowledge, this is the first report which exploits fractionation techniques to establish the existing relationship between *S. sipylea* phytochemical composition and biological activities. Furthermore, until now, only two studies have phytochemically analyzed *S. sipylea* methanolic extracts [11,22]. In this regard, Tomou et al. [11] have found that *S. sipylea* methanolic extract mainly contained flavones (e.g., apigenin, isoscutellarein, and their derivatives), phenylethanoid glycosides (e.g., martynoside), and iridoids (e.g., ajugoside, melittoside), highlighting a phytochemical pattern similar to that reported by Axiotis et al. [22]. Moreover, these results agree with previous literature evidence on *Sideritis* spp. Indeed, Petreska et al. [35] showed that *Sideritis* spp. from the Balkan Peninsula, such as *S. scardica*, *S. raeseri*, *S. taurica*, *S. syriaca*, and *S. perfoliate*, share a similar phytochemical composition distinguished by the presence of phenylethanoid glycosides and flavonoid acetylglycosides, which account for the 90% of all phenolic compounds [36]. In this context and considering our results, it also seems that *S. sipylea* possesses a phytocomplex similar to that of related taxa, although further quantitative analysis is required to better define it.

Afterward, the radical scavenger properties of the fractions against DPPH, ABTS, and NO radicals were evaluated by a test battery; indeed, free radicals are thought to be responsible for several pathologies such as chronic inflammation and oxidative damage to cellular structures leading, among others, to the development of cancer diseases [36]. Under our experimental conditions, all the samples were able to scavenge DPPH and ABTS radicals, although S10 and S20 were the most active by exhibiting IC_50_ values almost half that of S50. Conversely, only S10 scavenged NO radicals, although with less potency than the other ones.

DPPH and ABTS are synthetic radicals that can be neutralized by electron- or hydrogen-transfer mechanisms, although they differ in specificities and kinetics. Indeed, DPPH is scavenged by small molecules, due to the steric hindrance at the radical site, while ABTS is able to react with both lipophilic and hydrophilic compounds due to poor selectivity [33]. NO is a species released by cells during stress and a marked increase in its levels is involved in the onset of inflammation [37].

In line with our results, other studies have highlighted radical scavenger properties, especially against DPPH radical, of *S. sipylea* methanolic extracts. Particularly, a previous study showed that the methanolic extract was the most effective in neutralizing DPPH (IC_50_ 115 µg/mL) compared to the other samples [22]. Furthermore, Nakiboglu et al. [26] have found a strong antioxidant activity of both ethanolic and methanolic extracts from aerial parts of *S. sipylea*, with IC_50_ values equal to 50 µg/mL and 70 µg/mL, respectively. At last, Tomou et al. [11] have highlighted a lower scavenging power of *S. sipylea*, with IC_50_ values of about 595 µg/mL and 303 µg/mL towards DPPH and ABTS, respectively. Regarding NO, no studies were carried out on *S. sipylea*, although another related species, namely *Sideritis perfoliate*, displayed a moderate nitric oxide scavenging activity similar to that of S10 fraction (266.0 ± 7.1 µg/mL IC_50_ value) [37].

Based on this evidence, the radical scavenging abilities of the fractions, especially S10, seem to be ascribable to the presence of both hydrophilic and lipophilic phytochemicals, such as phenolic acids, flavones, and phenylethanoid glycosides, for which previous evidence has been reported [36]. However, further studies are required to clarify their involvement in the observed activity.

The fractions have also been evaluated for their ability to chelate or reduce iron ions, and to inhibit the production of advanced glycation end products (AGEs), both considered indirect oxidative mechanisms. Indeed, ROS generation can be facilitated by the presence of iron species, which are involved in the Fenton reaction; moreover, also AGEs are responsible for oxidative stress production [28]. Therefore, both metal chelating/reducing ability and AGE inhibition can provide benefits in pathological conditions, blocking oxidative stress and the consequent inflammatory damage.

In the present study, only the S10 fraction exhibited a moderate chelating power, while all the samples were able to reduce ferric ions, S10 and S20 being the most effective. Regarding the AGE formation inhibition, all the samples showed moderate activity, although the maximum effect ranging between 38% and 50% was achieved only at the highest tested concentration.

While until now, no studies have been conducted to evaluate the ability of *Sideritis* spp. to inhibit AGE formation, previous evidence has reported their ferric reducing power. Particularly, Tomou et al. [11] highlighted a low effect of the *S. sipylea* methanolic extract, with a 508 µg/mL IC_50_ value. Furthermore, Lytra et al. [38] showed that the methanolic extract of *S. cypria* was able to reduce ferric ions with an IC_50_ value of 793.45 mg trolox/g dry material. As for the radical scavenger properties, in this case, the phenolic derivatives also seem to be responsible for the antioxidant activity. Considering the highest activity of S10 and S20 fractions, it is likely that fractionation led to their enrichment in bioactive compounds. For example, it was reported that lavandulifolioside possesses antioxidant properties; moreover, it has been reported that the presence of O-dihydroxyphenyl groups in phenylethanoid glycosides enhances their antioxidant capacity [38].

Considering the results obtained in colorimetric assays and deeply investigating the biological properties of our samples, cytoprotective studies were carried out by using tBOOH as an inducer of oxidative stress. Particularly, preliminary cytotoxicity experiments were performed to choose nontoxic concentrations to be further tested. Moreover, the nonmalignant RAW264.7 (mouse macrophages), BEAS-2B (human bronchial epithelium cells), and H69 (human intrahepatic cholangiocytes) cell lines were used as a model, and the *Sideritis* spp. extracts are traditionally used to enforce the immune system and to relieve several ailments, among which respiratory and gastrointestinal ones [11,22,29]. Furthermore, both MTT and Neutral Red assays were performed to evaluate possible protection from the damage at the mitochondrial or lysosomal level. TBOOH is a pro-oxidative agent metabolized to free radicals, such as peroxyl and alkoxyl radicals, by cytochrome P450. Although its deleterious effect is mainly direct to mitochondria, some studies have highlighted that it is also able to indirectly affect lysosomal functionality by impairing the autophagic flux [30].

Under our experimental conditions, no cytoprotective effects were observed in BEAS-2B cells. Conversely, all the samples were able to counteract the tBOOH-induced oxidative damage in both RAW264.7 and H69 cell lines at nontoxic concentrations. Particularly, the highest cytoprotection was highlighted in H69 cells, especially for S10, by the MTT assay, allowing us to hypothesize that our fractions were able to protect mitochondria from oxidative damage. This is an important finding considering that mitochondria represent the main target of ROS attack. Moreover, dysfunctional mitochondria are also responsible for excessive ROS generation. This creates a vicious cycle that results in sustained ROS production that leads to oxidative stress and finally to cell death [30].

The deep investigation conducted in H69 cells also highlighted that our fractions were able to reduce the ROS levels induced by tBOOH by affecting GSH production. Indeed, tBOOH is detoxified by GSH through a reaction catalyzed by glutathione peroxidase, which finally led to the production of t-butanol and glutathione disulfide (GSSG) [39]. Accordingly, our results showed that the most potent fraction, namely S10, determined an increase of both GSH and GSSG levels in the presence of tBOOH; conversely, the other samples increased the GSSG production without affecting GSH one. These results allow us to hypothesize that while the GSH antioxidant defense system is the primary target of S10, other mechanisms could be involved in the antioxidant activity of S20 and S50.

Few reports have investigated the link between the cytoprotective properties of *Sideritis* spp. and GSH, some of which focused on kaurene diterpenes, which were not present in our fractions, or on non-characterized extracts [40,41,42]. However, Vasilopoulou et al. [43] have reported that the consumption of herbal tea from *Sideritis clandestina* determined an increase in GSH levels in mouse brain, so conferring antioxidant protection to rodent tissues. The specific constituent responsible for this activity was not identified; however, the authors hypothesized that derivatives of quinic acid, melittoside, and apigenin could be involved, as they are the main compounds of their extract and are extensively studied in the literature. Conversely, another study highlighted an inhibition of glutathione reductase by hypolaetin, isoscutellarein, and 3′-hydroxy-4′-*O*-methylisoscutellarein, isolated from aerial parts of *Sideritis brevibracteata* [44]. However, this evidence disagrees with our results. Indeed, glutathione reductase is responsible for the reduction of glutathione disulfide (GSSG) to its thiol form GSH; consequently, its inhibition may affect the cellular defense system and cause cellular oxidative stress, which was not observed in the presence of our samples.

## 4. Materials and Methods

### 4.1. Chemicals and Reagents

All the solvents used for extraction were of analytical grade. More specifically, dichloromethane (CH_2_Cl_2_), methanol (MeOH), and ethyl acetate (EtOAc) were purchased from Merk (Darmstadt, Germany). Water (H_2_O) was used after distillation. Acetonitrile and formic acid of LC-MS grade were acquired from Fischer Scientific (Leicestershire, UK) and ultrapure water obtained from a Milli-Q^®^ purification system (Merck Millipore, Darmstadt, Germany) was used for LC-MS analysis as well as to prepare all aqueous solutions. Amberlite XAD-7 resin (Rohm and Hass, Paris, France, Europe) was employed for the initial treatment of the methanol extract. Silica gel 60 H (Merk, Darmastadt, Germany) for the vacuum liquid chromatography (VLC) column was used.

All the chemicals, including Folin-Ciocalteu’s phenol reagent, tannic acid (Ph Eur purity), aluminum chloride hexahydrate (AlCl_3_ × 6H_2_O; Ph Eur purity), 1,1-diphenyl-2-picrylhydrazyl radical (DPPH; 95% purity), 2,2-azino-bis (3-thylbenzothiazoline-6-sulfonic acid) diammonium salt (ABTS; 98% purity), 2,2-azobis (2-methylpropionamidine) dihydrochloride (AAPH; 97% purity), ferrozine (97% purity), hydroxylamine hydrochloride (98% purity), iron (III) chloride (FeCl_3_ × 6H_2_O; 97% purity), iron (II) sulfate heptahydrate (FeSO_4_ × 7H_2_O; 99% purity), trolox (97% purity), quercetin (98% purity), rutin (99% purity), tannic acid (98% purity), bovine serum albumin, glucose, fructose, sodium azide, iron (II) chloride (FeCl_2_ × 4H_2_O; 99% purity), polyvinylpyrrolidone (PVP), sodium carbonate, sodium nitroprusside, Griess reagent, and tert-butyl hydroperoxide (tBOOH; 80% purity) were purchased from Merck (Darmstadt, Germany). Dulbecco’s Modified Eagle’s Medium (DMEM), RPMI 1640 medium, and fetal bovine serum were provided by Aurogene (Rome, Italy).

### 4.2. Plant Material and Extraction Procedure

The sample of *Sideritis sipylea* Boiss. was provided from local producers from North Aegean Island of Greece, Lesvos, collected from Olympus mt., ca. 800 m, and was authenticated by Dr. Makis Axiotis. Voucher specimens were deposited in the herbarium of the Faculty of Pharmacy, Department of Pharmacognosy and Natural Products Chemistry, National and Kapodistrian University of Athens, Greece. The aerial parts were dried in a well-ventilated, dark place at room temperature and stored in glass bottles. Then, the air-dried material was powdered in a mill and a portion (104 g) thereof was subjected to ultrasound-assisted extraction (UAE) with 1 L × 3 MeOH at room temperature by UAE at 40 °C for 1 h. Then, the extract was filtered, lyophilized, and then weighted and stored at 4 °C before analysis. The weight of the final extract was 55.52 g (yielded 53.4% *w*/*w*). In order to selectively isolate the phenolic fraction of the MeOH extract of *S. sipylea* adsorption, resin column chromatography was used. Specifically, resin Amberlite^®^ XAD-7 from Sigma Aldrich (Steinheim, Germany) was conditioned with an excess of distilled H_2_O and swollen into MeOH overnight. The excess MeOH was removed by rinsing the swollen adsorbent with distilled H_2_O. A total of 20 g of MeOH extract diluted in distilled H_2_O was applied to a separating funnel containing 200 mg of Amberlite^®^ XAD-7 resin, rinsed with distilled H_2_O and the water-soluble fraction was collected. The phenolics adsorbed by the resin were eluted using MeOH.

The enriched fraction (6.2 g, yielded 31% *w*/*w*) was evaporated in vacuo to dry and was subjected subsequently to a vacuum liquid chromatography (VLC) on silica gel and eluted by increasing polarity of solvent systems (CH_2_Cl_2_/EtOAc/MeOH) to give eight fractions (Supplementary Material Table S1). Fraction S10 (932.6 mg), fraction S20 (1576.1 mg), and fraction S50 (1451.1 mg) were further analyzed by UHPLC-HRMS and HRMS/MS analysis.

### 4.3. Phytochemical Analysis

#### 4.3.1. Determination of Total Polyphenols, Tannins, and Flavonoids

The Folin–Ciocalteu and the aluminum chloride spectrophotometric methods were applied to determine the total amount of polyphenols, tannins, and flavonoids, according to Di Sotto et al. [28]. For both polyphenols and tannins, the absorbance was measured at 765 nm and the results were expressed as tannic acid equivalents (TAEs) per gram of sample. Instead, total flavonoids were measured at 415 nm, and the amount was expressed as quercetin equivalents (QE) per gram of sample. Equations of calibration curves for tannic acid and quercetin, calculated by linear regression (GraphPad Prism™ 8.0.1), were Y = 0.03910X + 0.02627 (r^2^ = 0.980) and Y = 0.002351X + 0.009454 (r^2^ = 0.988), respectively.

#### 4.3.2. Ultra-Performance Liquid Chromatography Coupled with High-Resolution Mass Spectrometry (UHPLC-HRMS)

UHPLC-HRMS analyses were performed using an Acquity H-Class UPLC system (Waters Corp., Milford, CT, USA), equipped with a quaternary pump, an autosampler, an online vacuum degasser, and a temperature-controlled column and sample compartment, hyphenated to a hybrid LTQ-Orbitrap Discovery XL (Thermo Scientific, Brehmen, Germany) mass spectrometer with an electrospray ionization (ESI) source. Xcalibur 2.0.7 (Thermo Scientific) software was used for data acquisition and processing. For all analyses, the samples were diluted at a concentration of 200 μg/mL, using MeOH/H_2_O 60:40 (*v*/*v*) for the methanolic extract. A Fortis C18 (Fortis Technologies Ltd., Cheshire, UK) column (100 × 2.1 mm, 1.7 μm) was used with a mobile phase consisting of water with 0.1% formic acid (solvent A) and acetonitrile (solvent B). The gradient program was as follows: initially, 5% (B) was maintained for 3 min, then increased until reaching 100% (B) in 21 min, maintained at 100% (B) for 3 min, and finally decreased to 5% (B) in 2 min and held at initial conditions (5 min) for re-equilibration, with a total run time of 30 min. The flow rate was 0.4 mL/min, while the injection volume was 10 μL. The column temperature was set to 40 °C. MS data acquisition was performed in negative (ESI-) ionization modes, in the full scan mass range of *m*/*z* 115.0–1000.0, using a resolution of 30,000. The capillary temperature was set at 350 °C. The tuning of capillary voltage and tube lens was −30 and −100 V in negative mode. The source voltage was set at 2.70 kV (ESI-), while the source current was 100 μA.

MS^2^ spectra were recorded by selecting data-depending acquisition, with the collision-induced dissociation (CID) value at 35% and a mass resolution of 7500. Nitrogen was used as sheath gas and auxiliary gas, with a flow rate set at 40 and 10 arbitrary units, respectively.

### 4.4. Radical Scavenging Activity

The ability of the samples to scavenge DPPH and ABTS radicals was determined according to the previously described spectrophotometric methods [33]. The scavenger activity of the samples towards nitric oxide radicals was investigated too, according to Fraisse et al. [45], with minor changes. Briefly, a sodium nitroprusside (SNP) solution (100 µL; 3.125 mM) was mixed with different concentrations of the test samples (25 µL) and incubated for 180 min in the dark at room temperature. A reaction mixture without extract or containing trolox was also prepared to be employed as negative and positive controls, respectively. Then, 100 µL of Griess reagent (1% sulfanilamide, 5% phosphoric acid, and 0.1% NED) were added to the reaction mixtures. Absorbance was recorded at 546 nm, after 10 min incubation, by using a microplate reader (Epoch Microplate Spectrophotometer, BioTeK^®^ Instruments Inc., Winooski, VT, USA).

For all assays, results were expressed as a percentage of scavenger activity, calculated as follows: 100 × (A_control_ − A_sample_)/A_control_, where A_control_ is the absorbance of the radical alone, while A_sample_ is that of radical with the sample.

### 4.5. Iron Chelating and Reducing Activity

The ability of the samples to chelate and reduce ferrous and ferric ions was evaluated by the ferrozine assay according to previously published methods [46].

Particularly, the chelating activity was assessed against both ferrous and ferric ions. In the first case, FeSO_4_ × 7H_2_O (50 µL; 200 µM) was mixed for 2 min with the samples (50 µL) and acetate buffer (50 µL; 0.1 M, pH = 4.5), while ferric ion chelation was evaluated by mixing FeCl_3_ × 6H_2_O (50 µL; 200 µM) with the samples (50 µL) and hydroxylamine (50 µL; 5 mM). Then, ferrozine solution (50 µL; 5 mM) was added to both mixtures and the absorbance was measured at 562 nm. The percentage of chelating activity was calculated as follows: 100 × (A_control_ − A_sample_)/A_control_, where A_control_ is the absorbance of the vehicle while A_sample_ is that of the tested sample. In each experiment, the positive controls rutin and quercetin were included too.

Regarding ferric ion reducing activity, FeCl_3_ × 6H_2_O (50 µL; 200 µM) was blended with samples (50 µL) and acetate buffer (50 µL) for two minutes. Then, ferrozine solution (50 µL; 5 mM) was added and the absorbance was measured at 562 nm. The percentage of reducing activity was calculated as follows: 100 × (A_sample_ − A_control_)/A_control_, where A_control_ is the absorbance of the vehicle while A_sample_ is that of the tested sample. The ferric reducing power was evaluated in relation to the standard reference trolox.

### 4.6. Inhibition of Advanced Glycation End-Product (AGE) Formation

The ability of the samples (1–250 µg/mL) to block the AGE formation was measured according to Sissi et al. [33]. The standard antiglycation agent rutin was used as a positive control. Fluorescence was measured at an excitation wavelength of 355 nm and an emission of 460 nm. Results were expressed as a percentage of the control by using the following formula: (A_sample_/A_control_) × 100, where A_control_ is the fluorescence of the control, whereas A_sample_ is the fluorescence of the sample.

### 4.7. Cytoprotective Activity under Oxidative Stress

#### 4.7.1. Cell Culture

The nonmalignant BEAS-2B (human bronchial epithelium cells), H69 (human intrahepatic cholangiocytes), and RAW264.7 (mouse macrophages) cell lines were used as a model to study the protective effects of tested samples. Particularly, BEAS-2B was obtained from the American Type Culture Collection (ATCC), while H69 and RAW264.7 were a kind gift from Romina Mancinelli (Department of Anatomical, Histological, Forensic and Orthopedic Sciences, Sapienza University of Rome, Italy) and Professor Lucia Nencioni (Department of Public Health and Infectious Diseases, Istituto Pasteur Italia-Fondazione Cenci-Bolognetti, Sapienza University, Italy).

Cell lines were grown under standard conditions (37 °C and 5% CO_2_) according to previously published methods [47,48]. Subculturing was made every 4 days, and the growth media were renewed twice a week, as recommended by the supplier. All experiments were performed when cells reached the logarithmic growth phase.

#### 4.7.2. Cytotoxicity Assay

To perform the experiments, the cultured cells were seeded into 96-well microplates (2 × 10^4^ cells/well) and allowed to grow for 24 h; then, progressive dilutions of *S. sipylea* fractions in DMSO (100% *v*/*v*) were added to them. A maximum DMSO concentration of 1% (*v*/*v*) was used in the cell medium to avoid any solvent toxicity. Cell viability was measured after 24 h of incubation with the samples by the 3-[4,5-dimethylthiazol-2-yl]-2,5-diphenyl tetrazolium bromide (MTT) and Neutral Red (NR) assays, according to previously published methods [28,49]. The results were expressed as a percentage of the vehicle control. A treatment was considered cytotoxic when the cell viability was less than 70% with respect to the control [50].

#### 4.7.3. Cytoprotection towards the Oxidative Damage Induced by Tert-Butyl Hydroperoxide (tBOOH)

To evaluate the cytoprotective activity of *S. sipylea* fractions towards the pro-oxidant agent tBOOH, 2 × 10^4^ cells were seeded into 96-well microplates and allowed to grow for 24 h. Then, confluent cells (about 60–70% of the confluence) were pretreated for 24 h with progressive nontoxic concentrations of the samples (10–250 µg/mL). Afterward, a low-toxic concentration (about 40% cytotoxicity as found in preliminary experiments) of tBOOH (5 μM) was added for 3 h to cells, and then the cell viability was measured as described above (see paragraph 4.7.2).

The same pretreatment protocol was also used to determine some oxidative stress parameters, namely the intracellular levels of reactive oxygen species (ROS) and glutathione, as follows.

#### 4.7.4. Determination of Intracellular Levels of Reactive Oxygen Species (ROS)

The ability of the samples to counteract the ROS generation induced by tBOOH treatment was measured by the 2,7-dichlorofluorescein diacetate assay (DCFH-DA), according to Di Giacomo et al. [51] with slight changes. In each experiment, a vehicle control (corresponding to the basal ROS level) and positive control (corresponding to the highest oxidation) were included too. The DCF fluorescence was measured at an excitation wavelength of 485 nm and emission wavelength of 528 nm by using the Cytation 1 Cell Imaging Multimode Reader (BioTeK^®^ Instruments Inc., Winooski, VT, USA). Fluorescence intensity was determined by Gen5™ Microplate Reader and Imager Software 3.11 and normalized with respect to cell number. Results were expressed as oxidation index, corresponding to the ratio between the DCF fluorescence of the sample and vehicle control.

#### 4.7.5. Chromatographic Determination of Intracellular Glutathione Levels

Intracellular levels of reduced (GSH) and oxidized (GSSG) glutathione were measured by HPLC-UV, according to previously published methods [28]. Particularly, cell pellets (1 × 10^6^) were suspended in 10% ice-cold TCA and centrifuged for 15 min at 9000× *g*. The supernatant was collected, and GSH and GSSG were measured by HPLC with UV detection at 215 nm. The separation was achieved using an Infinity Lab poroshell 120 EC-C18 column (3 × 150 mm, 2.7 m) at a flow rate of 0.8 mL/min with the following elution gradient: 0–3 min 100% A + 0% B, 3–10 min from 100% A to 100% B. The composition of mobile phase A was 0.1% trifluoroacetic acid in water and mobile phase B was 0.1% trifluoroacetic acid in water/acetonitrile (93:7). Under our chromatographic conditions, retention times were 2.58 min and 7.01 min for GSH and GSSG, respectively.

### 4.8. Statistical Analysis

Data from at least three experiments, each one including at least three technical replicates per treatment, were pooled and analyzed by GraphPad Prism™ (Version 8.00) software (GraphPad Software, Inc., San Diego, CA, USA) and expressed as mean ± standard error (SE). The one-way analysis of variance (one-way ANOVA), followed by Dunnett’s multiple comparison post-test, was used to analyze the difference between treatments. Instead, unpaired data were analyzed with Student’s *t*-test. A response was considered statistically significant when a *p*-value < 0.05 was obtained with respect to the control. The concentration–response curves were constructed using the Hill equation [28].

## 5. Conclusions

Altogether, the present results support the traditional use of *S. sipylea*, especially for gastrointestinal ailments. Indeed, oxidative stress has been implicated in cholestasis disease, which is characterized by an impairment of biliary acid secretion and consequently by digestive disorders [52]. The strong antioxidant activity highlighted by the different fractions in our study allows us to hypothesize that the samples can counteract oxidative stress through direct and indirect antioxidant mechanisms, and by reinforcing the cellular antioxidant defense system. However, it remains to define the main bioactive compounds involved in the observed biological properties, without excluding possible synergistic interactions among the different phytochemicals of the entire phytocomplex. Moreover, a deep characterization of the pharmacological mechanism is needed. Further studies will help us to better clarify these critical points in order to exploit *S. sipylea* fractions for nutraceutical and pharmaceutical purposes.

## Figures and Tables

**Table 1:** Total amount of polyphenols, tannins, and flavonoids in S10, S20, and S50 fractions from *Sideritis sipylea* Boiss. Data are expressed as means ± standard error (SE) of at least two experiments and six replicates.

*Sideritis sipylea* Fractions	Total Polyphenols	Tannins	Flavonoids
	μg TAE/mg		μg QE/mg
S10	72.03 ± 0.02	31.07 ± 0.02	2216.40 ± 25.03
S20	70.03 ± 0.01	28.07 ± 0.02 *	903.20 ± 15.02 ***
S50	58.04 ± 0.01 ***°°°	29.07 ± 0.01	746.40 ± 17.01 ***°°°

TAE: tannic acid equivalents; QE: quercetin equivalents; * *p* < 0.01 and *** *p* < 0.001 vs. S10 fraction (Student’s *t*-Test); °°° *p* < 0.001 vs. S20 fraction (Student’s *t*-Test).

**Table 2:** Chemical components of S10, S20, and S50 fractions from the methanol total extract of wild *S. sipylea* identified by UPLC-ESI (-)-HRMS.

Νο.	Compound	t_R_ (min.)	[M-H]^−^ m/z	Suggested Formula	Area (×10^7^)			Ref.
					**S10**	**S20**	**S50**	
1	Melittoside derivative	0.90	569.1726	C_22_H_33_O_17_	0.2	1.5	1.8	[21]
2	Quinic acid	1.04	191.0568	C_7_H_11_O_6_	-	-	**4.7**	[21]
3	Feruloylquinic acid	1.11	367.1037	C_17_H_19_O_9_	0.3	-	-	[21]
4	Melittoside derivative	1.46	569.1726	C_22_H_33_O_17_	0.1	2.1	**3.5**	[21]
5	Chlorogenic acid	7.23	353.0880	C_16_H_17_O_9_	-	-	**5.4**	[21]
6	Iridoid derivative	8.42	435.1491	C_18_H_27_O_12_	**14.6**	1.1	0.3	[26]
7	Echinacoside	9.60	785.2509	C_35_H_45_O_20_	-	**3.7**	0.9	[21]
8	Lavandulifolioside	9.70	755.2393	C_34_H_43_O_19_	0.3	**10.3**	1.7	[26]
9	Isoverbascoside	9.90	623.1989	C_29_H_35_O_15_	**8.4**	**5.3**	1.9	[21]
10	Isoscutelarein 7-*O*-allosyl-(1→2)-glucoside	10.24	609.1450	C_27_H_29_O_16_	1.3	0.7	-	[21]
11	Leucoseptoside A	10.77	637.2142	C_30_H_37_O_15_	0.2	0.1	tr	[21]
12	Apigenin 7-*O*-glucoside	10.90	431.0984	C_21_H_19_O_10_	0.9	0.6	0.2	[21]
13	Luteolin 7-*O*-allosyl-(1→2)-[6″-*O*-acetyl]-glucoside	11.27	651.1570	C_29_H_31_O_17_	**8.2**	1.3	-	[21]
14	4′-*O*-methylisoscutellarein 7-*O*-allosyl-(1→2)-glucoside	11.88	623.1625	C_28_H_31_O_16_	**8.1**	**4.8**	0.2	[21,26]
15	4′-*O*-methylisoscutellarein 7-*O*-allosyl-(1→2)-[6″-*O*-acetyl]-glucoside	12.94	665.1728	C_30_H_33_O_17_	**23.7**	**8.3**	1.4	[21,26]
16	Apigenin 7-(6″-*p*-coumaroylglucoside)	13.11	577.1356	C_30_H_25_O_12_	1.1	0.7	0.3	[21,26]
17	Apigenin 7-(4″-*p*-coumaroylglucoside)	13.90	577.1356	C_30_H_25_O_12_	3.3	1.2	0.8	[21,26]
18	Apigenin	14.20	269.0400	C_15_H_9_O_5_	tr	tr	tr	[21,26]
19	4′-*O*-Methylisoscutellarein 7-*O*-[6″′-*O*-acetyl]-allosyl-(1→2)-[6″-*O*-acetyl]-glucoside	14.88	707.1808	C_32_H_35_O_18_	-	tr	tr	[21]

Tr: trace.

**Table 3:** IC_50_ values (µg/mL) of S10, S20, and S50 fractions from *Sideritis sipylea* Boiss and the positive controls for radical scavenging activity, iron chelating, and reducing effect, and inhibition of advanced glycation end products (AGE).

Assay	IC_50_ (CL ^a^) µg/mL			
	S10	S20	S50	Positive Control
DPPH scavenging activity	156.7 (116.3–211.2)	150.3 (133.2–169.6)	324.7 (262.7–401.3) *°	4.9 (3.67–6.46) ^b^
ABTS scavenging activity	33.5 (25.2–44.5)	41.8 (23.7–73.9)	73.1 (67.9–78.6) **	2.1 (1.8–2.4) ^b^
NO scavenging activity	204.4 (108.5–396.6)	ne ^c^	ne	85.4 (80.6–87.4) ^b^
Ferrous ion chelating activity	188.9 (147.5–280.1)	ne	ne	51.1 (32.9–61.7) ^d^
Ferric ion chelating activity	273.0 (255.5–294.0)	ne	ne	45.2 (13.1–75.5) ^d^
Ferric ion reducing activity	30.33 (25.22–36.19)	17.68 (15.03–20.82) *	87.82 (74.36–126.8) *°°	4.3 (3.1–5.5) ^b^
AGE formation inhibition	ne	ne	ne	3.2 (2.7–3.6) ^e^

^a^ CL: confidence limit; ^b^ trolox; ^c^ ne: not evaluable being the achieved effect lower than 80%; ^d^ quercetin, ^e^ rutin. * *p* < 0.05 and ** *p* < 0.01 vs. S10 (Student *t*-test); ° *p* < 0.05 and °° *p* < 0.01 vs. S20 (Student *t*-test).

**Table 4:** Levels of GSH and GSSG after treatment with S10, S20, and S50 fractions from *Sideritis sipylea* Boiss, the pro-oxidant agent tBOOH and their combination in H69 cells. GSH and GSSH were evaluated in cell lysates, calculated with respect to the corresponding calibration curves, and normalized on cell viability. Data are mean ± SE from at least three independent experiments (*n* = 3). °°° *p* < 0.001 vs. control, while ** *p* < 0.01 and *** *p* < 0.001 vs. tBOOH (one-way ANOVA followed by Dunnett’s multiple comparison post-test).

Treatment	GSH	GSSG
	(µM)	
Control	147.0 ± 1.8	48.2 ± 0.1
tBOOH [5 µM]	120.7 ± 2.2 °°°	158.3 ± 3.3 °°°
*S. sipylea* 10 [100 µg/mL]	167.3 ± 1.5 °°°	60.9 ± 2.2 °°°
*S. sipylea* 20 [100 µg/mL]	80.5 ± 1.7 °°°	49.0 ± 0.3
*S. sipylea* 50 [100 µg/mL]	92.4 ± 2.0 °°°	50.2 ± 0.3
*S. sipylea* 10 [100 µg/mL] + tBOOH [5 µM]	179.6 ± 4.0 ***	70.0 ± 3.6 ***
*S. sipylea* 20 [100 µg/mL] + tBOOH [5 µM]	104.0 ± 1.8 **	63.9 ± 0.2 ***
*S. sipylea* 50 [100 µg/mL] + tBOOH [5 µM]	119.8 ± 3.1	73.7 ± 1.6 ***

## Data Availability

Data is contained within the article and Supplementary Materials.

## Supplementary Materials

The following supporting information can be downloaded at: https://www.mdpi.com/article/10.3390/ph15080987/s1, Table S1: *S. sipylea* methanolic enriched extract fractions of increased polarity in mass (g) and their % (*w*/*w*) yields; Figure S1: Chemical structures of the identified compounds from the selected fractions S10, S20, and S50 respectively from wild *S. sipylea* Boiss.; Figure S2: UPLC-ESI (-)-HRMS chromatograms of (i) EtOAc/MeOH (10%) (S10), (ii) EtOAc/MeOH (20%) (S20), and (iii) EtOAc/MeOH (50%) (S50) fractions of the methanol extract from wild *S. sipylea*; Figure S3: AGE formation inhibition by the dried fractions from *S. sipylea* Boiss.

## References

[1] Davis P. (1982). Flora of Turkey and the East Aegean Islands.

[2] Güvenç A., Houghton P.J., Duman H., Coşkun M., Şahin P. (2005). Antioxidant Activity Studies on Selected Sideritis Species Native to Turkey. Pharm. Biol..

[3] González-Burgos E., Carretero M.E., Gómez-Serranillos M.P. (2011). *Sideritis* Spp.: Uses, Chemical Composition and Pharmacological Activities—A Review. J. Ethnopharmacol..

[4] Strid A., Kit T. (1991). Mountain Flora of Greece.

[5] Axiotis E., Halabalaki M., Skaltsounis L.A. (2018). An Ethnobotanical Study of Medicinal Plants in the Greek Islands of North Aegean Region. Front. Pharmacol..

[6] Tsioutsiou E.E., Giordani P., Hanlidou E., Biagi M., De Feo V., Cornara L. (2019). Ethnobotanical Study of Medicinal Plants Used in Central Macedonia, Greece. Evid. Based Complement. Altern. Med..

[7] Vokou D., Katradi K., Kokkini S. (1993). Ethnobotanical Survey of Zagori (Epirus, Greece), a Renowned Centre of Folk Medicine in the Past. J. Ethnopharmacol..

[8] Petrakou K., Iatrou G., Lamari F.N. (2019). Ethnopharmacological Survey of Medicinal Plants Traded in Herbal Markets in the Peloponnisos, Greece. J. Herb. Med..

[9] Gürbüz I., Özkan A.M., Yesilada E., Kutsal O. (2005). Anti-Ulcerogenic Activity of Some Plants Used in Folk Medicine of Pinarbasi (Kayseri, Turkey). J. Ethnopharmacol..

[10] Aboutabl E.A., Nassar M.I., Elsakhawy F.M., Maklad Y.A., Osman A.F., El-Khrisy E.A.M. (2002). Phytochemical and Pharmacological Studies on *Sideritis taurica* Stephan Ex Wild. J. Ethnopharmacol..

[11] Tomou E.M., Lytra K., Chrysargyris A., Christofi M.D., Miltiadous P., Corongiu G.L., Tziouvelis M., Tzortzakis N., Skaltsa H. (2021). Polar Constituents, Biological Effects and Nutritional Value of *Sideritis sipylea* Boiss. Nat. Prod. Res..

[12] Basile A., Senatore F., Gargano R., Sorbo S., Del Pezzo M., Lavitola A., Ritieni A., Bruno M., Spatuzzi D., Rigano D. (2006). Antibacterial and Antioxidant Activities in *Sideritis italica* (Miller) Greuter et Burdet Essential Oils. J. Ethnopharmacol..

[13] Aligiannis N., Kalpoutzakis E., Chinou I.B., Mitakou S., Gikas E., Tsarbopoulos A. (2001). Composition and Antimicrobial Activity of the Essential Oils of Five Taxa of *Sideritis* from Greece. J. Agric. Food Chem..

[14] Dulger B., Gonuz A., Aysel V. (2006). Inhibition of Clotrimazole-Resistant Candida Albicans by Some Endemic Sideritis Species from Turkey. Fitoterapia.

[15] Todorova M., Trendafilova A. (2014). *Sideritis scardica* Griseb., an Endemic Species of Balkan Peninsula: Traditional Uses, Cultivation, Chemical Composition, Biological Activity. J. Ethnopharmacol..

[16] Hofrichter J., Krohn M., Schumacher T., Lange C., Feistel B., Walbroel B., Pahnke J. (2016). *Sideritis* Spp. Extracts Enhance Memory and Learning in Alzheimer’s β-Amyloidosis Mouse Models and Aged C57Bl/6 Mice. J. Alzheimer’s Dis..

[17] Knörle R. (2012). Extracts of *Sideritis scardica* as Triple Monoamine Reuptake Inhibitors. J. Neural Transm..

[18] Committee on Herbal Medicinal Products (HMPC) (2015). European Union Herbal Monograph on Sideritis scardica Griseb.; Sideritis Clandestina (Bory & Chaub) Hayek; Sideritis Raeseri Boiss. & Heldr.; Sideritis Syriaca, L., Herba.

[19] Charami M.-T., Lazari D., Kariotis A., Skaltsa H., Hadjipavlou-Litina D., Souleles C. (2008). Antioxidant and Antiinflammatory Activities of *Sideritis perfoliata* Subsp. *Perfoliata* (Lamiaceae). Phytother. Res..

[20] Gabrieli C.N., Kefalas P.G., Kokkalou E.L. (2005). Antioxidant Activity of Flavonoids from *Sideritis raeseri*. J. Ethnopharmacol..

[21] Aslan İ., Kılıç T., Gören A.C., Topçu G. (2006). Toxicity of Acetone Extract of *Sideritis trojana* and 7-Epicandicandiol, 7-Epicandicandiol Diacetate and 18-Acetylsideroxol against Stored Pests *Acanthoscelides obtectus* (Say), *Sitophilus granarius* (L.) and *Ephestia kuehniella* (Zell.). Ind. Crops Prod..

[22] Axiotis E., Petrakis E.A., Halabalaki M., Mitakou S. (2020). Phytochemical Profile and Biological Activity of Endemic *Sideritis sipylea* Boiss. in North Aegean Greek Islands. Molecules.

[23] Sargın S.A., Akçicek E., Selvi S. (2013). An Ethnobotanical Study of Medicinal Plants Used by the Local People of Alaşehir (Manisa) in Turkey. J. Ethnopharmacol..

[24] Sargin S.A., Selvi S., López V. (2015). Ethnomedicinal Plants of Sarigöl District (Manisa), Turkey. J. Ethnopharmacol..

[25] Loğoğlu E., Arslan S., Oktemer A., Sakõyan I. (2006). Biological Activities of Some Natural Compounds from *Sideritis sipylea* Boiss. Phytother. Res..

[26] Nakiboglu M., Urek R.O., Kayali H.A., Tarhan L. (2007). Antioxidant Capacities of Endemic *Sideritis sipylea* and *Origanum sipyleum* from Turkey. Food Chem..

[27] Forman H.J., Zhang H. (2021). Targeting oxidative stress in disease: Promise and limitations of antioxidant therapy. Nat. Rev. Drug Discov..

[28] Di Sotto A., Locatelli M., Macone A., Toniolo C., Cesa S., Carradori S., Eufemi M., Mazzanti G., Di Giacomo S. (2019). Hypoglycemic, Antiglycation, and Cytoprotective Properties of a Phenol-Rich Extract from Waste Peel of *Punica granatum* L. var. Dente di Cavallo DC2. Molecules.

[29] Sarikurkcu C., Locatelli M., Mocan A., Zengin G., Kirkan B. (2020). Phenolic Profile and Bioactivities of *Sideritis perfoliate* L.: The Plant, Its Most Active Extract, and Its Broad Biological Properties. Front. Pharmacol..

[30] Kang L., Xiang Q., Zhan S., Song Y., Wang K., Zhao K., Li S., Shao Z., Yang C., Zhang Y. (2019). Restoration of Autophagic Flux Rescues Oxidative Damage and Mitochondrial Dysfunction to Protect against Intervertebral Disc Degeneration. Oxid. Med. Cell. Longev..

[31] Li Z., Jiang T., Lu Q., Xu K., He J., Xie L., Chen Z., Zheng Z., Ye L., Xu K. (2020). Berberine attenuated the cytotoxicity induced by t-BHP via inhibiting oxidative stress and mitochondria dysfunction in PC-12 cells. Cell. Mol. Neurobiol..

[32] Aneva I., Zhelev P., Kozuharova E., Danova K., Nabavi S.F., Behzad S. (2019). Genus *Sideritis*, section *Empedoclia* in southeastern Europe and Turkey—Studies in ethnopharmacology and recent progress of biological activities. Daru.

[33] Sissi S., Di Giacomo S., Ferrante C., Angelini P., Macone A., Giusti A.M., Toniolo C., Vitalone A., Abdellah A., Larhsini M. (2022). Characterization of the Phytochemical Composition and Bioactivities of Anacyclus maroccanus Ball. and Anacyclus radiatus Loisel Aerial Parts: Preliminary Evidence for the Possible Development of Moroccan Plants. Molecules.

[34] Ibraliu A., Trendafilova B.A., Anđelković B.D., Qazimi B., Gođevac D.M., Shengjergji D., Bebeci E., Stefkov G., Zdunic G., Aneva I.I. (2015). Comparative Study of Balkan *Sideritis* Species from Albania, Bulgaria and Macedonia. Eur. J. Med. Plants.

[35] Petreska J., Stefkov G., Kulevanova S., Alipieva K., Bankova V., Stefova M. (2011). Phenolic compounds of mountain tea from the Balkans: LC/DAD/ESI/MSn profile and content. Nat. Prod. Commun..

[36] Żyżelewicz D., Kulbat-Warycha K., Oracz J., Żyżelewicz K. (2020). Polyphenols and Other Bioactive Compounds of *Sideritis* Plants and Their Potential Biological Activity. Molecules.

[37] Lall N., Chrysargyris A., Lambrechts I., Fibrich B., Blom Van Staden A., Twilley D., de Canha M.N., Oosthuizen C.B., Bodiba D., Tzortzakis N. (2019). *Sideritis Perfoliata* (Subsp. Perfoliata) Nutritive Value and Its Potential Medicinal Properties. Antioxidants.

[38] Lytra K., Tomou E.M., Chrysargyris A., Christofi M.D., Miltiadous P., Tzortzakis N., Skaltsa H. (2021). Bio-Guided Investigation of *Sideritis cypria* Methanol Extract Driven by in Vitro Antioxidant and Cytotoxic Assays. Chem. Biodivers..

[39] Jang H.I., Do G.M., Lee H.M., Ok H.M., Shin J.H., Kwon O. (2014). Schisandra chinensis Baillon regulates the gene expression of phase II antioxidant/detoxifying enzymes in hepatic damage induced rats. Nutr. Res. Pract..

[40] González-Burgos E., Carretero M.E., Gómez-Serranillos M.P. (2013). Kaurane diterpenes from *Sideritis* spp. exert a cytoprotective effect against oxidative injury that is associated with modulation of the Nrf2 system. Phytochemistry.

[41] González-Burgos E., Carretero M.E., Gómez-Serranillos M.P. (2013). Nrf2-dependent neuroprotective activity of diterpenoids isolated from *Sideritis* spp. J. Ethnopharmacol..

[42] Celik I., Kaya M.S. (2011). The antioxidant role of *Sideritis caesarea* infusion against TCA toxicity in rats. Br. J. Nutr..

[43] Vasilopoulou C.G., Kontogianni V.G., Linardaki Z.I., Iatrou G., Lamari F.N., Nerantzaki A.A., Gerothanassis I.P., Tzakos A.G., Margarity M. (2013). Phytochemical composition of “mountain tea” from *Sideritis clandestina* subsp. clandestina and evaluation of its behavioral and oxidant/antioxidant effects on adult mice. Eur. J. Nutr..

[44] Tandogan B., Güvenç A., Çalış İ., Ulusu N.N. (2011). In vitro effects of compounds isolated from Sideritis brevibracteata on bovine kidney cortex glutathione reductase. Acta Biochim. Pol..

[45] Fraisse D., Degerine-Roussel A., Bred A., Ndoye S.F., Vivier M., Felgines C., Senejoux F. (2018). A Novel HPLC Method for Direct Detection of Nitric Oxide Scavengers from Complex Plant Matrices and Its Application to *Aloysia triphylla* Leaves. Molecules.

[46] Di Sotto A., Vecchiato M., Abete L., Toniolo C., Giusti A.M., Mannina L., Locatelli M., Nicoletti M., Di Giacomo S. (2018). *Capsicum annuum* L. var. Cornetto di Pontecorvo PDO: Polyphenolic profile and in vitro biological activities. J. Funct. Foods.

[47] Di Sotto A., Gullì M., Acquaviva A., Tacchini M., Di Simone S.C., Chiavaroli A., Recinella L., Leone S., Brunetti L., Orlando G. (2022). Phytochemical and pharmacological profiles of the essential oil from the inflorescences of the *Cannabis sativa* L. Ind. Crops Prod..

[48] Checconi P., Salzano S., Bowler L., Mullen L., Mengozzi M., Hanschmann E.M., Lillig C.H., Sgarbanti R., Panella S., Nencioni L. (2015). Redox proteomics of the inflammatory secretome identifies a common set of redoxins and other glutathionylated proteins released in inflammation, influenza virus infection and oxidative stress. PLoS ONE.

[49] Zagórska-Dziok M., Bujak T., Ziemlewska A., Nizioł-Łukaszewska Z. (2021). Positive Effect of *Cannabis sativa* L. Herb Extracts on Skin Cells and Assessment of Cannabinoid-Based Hydrogels Properties. Molecules.

[50] Di Giacomo S., Mariano A., Gullì M., Fraschetti C., Vitalone A., Filippi A., Mannina L., Scotto d’Abusco A., Di Sotto A. (2021). Role of Caryophyllane Sesquiterpenes in the Entourage Effect of Felina 32 Hemp Inflorescence Phytocomplex in Triple Negative MDA-MB-468 Breast Cancer Cells. Molecules.

[51] Di Giacomo S., Abete L., Cocchiola R., Mazzanti G., Eufemi M., Di Sotto A. (2018). Caryophyllane sesquiterpenes inhibit DNA-damage by tobacco smoke in bacterial and mammalian cells. Food Chem. Toxicol..

[52] Wu W., Li K., Ran X., Wang W., Xu X., Zhang Y., Wei X., Zhang T. (2022). Combination of resveratrol and luteolin ameliorates α-naphthylisothiocyanate-induced cholestasis by regulating the bile acid homeostasis and suppressing oxidative stress. Food Funct..

