# Supplementary material for: Phytochemical Composition and Cytoprotective Properties of the Endemic Sideritis sipylea Boiss Greek Species: A Valorization Study
Source: Pharmaceuticals (Basel). 2022 Aug 11;15(8):987. doi: 10.3390/ph15080987 (PMC9414158; doi:10.3390/ph15080987)
Supplement: Supplementary file 1 [file pharmaceuticals-15-00987-s001.zip › pharmaceuticals-1827516-supplementary.pdf]

*pharmaceuticals*

*Supplementary material*

# Phytochemical Composition and Cytoprotective Properties of the Endemic *Sideritis sipylea* Boiss Greek Species: A Valorization Study

**Silvia Di Giacomo [1], Antonella Di Sotto [1,*], Apostolis Angelis [2], Ester Percaccio [1], Annabella Vitalone [1], Marco Gullì [1], Alberto Macone [3], Evangelos Axiotis [2,4,†] and Alexios Leandros Skaltsounis [2,4,†]**

[1] Department of Physiology and Pharmacology "V. Erspamer", Sapienza University of Rome, P.le Aldo Moro 5, 00185 Rome, Italy
[2] Department of Pharmacognosy and Natural Products Chemistry, Faculty of Pharmacy, National and Kapodistrian University of Athens, 15771 Athens, Greece
[3] Department of Biochemical Science "A. Rossi Fanelli", Sapienza University of Rome, P.le Aldo Moro 5, 00185 Rome, Italy
[4] Natural Products Research Center "NatPro Aegean", Gera, 81106 Lesvos, Greece
* Correspondence: antonella.disotto@uniroma1.it
† These authors equally contributed to the work.

**Table S1.** *S. sipylea* methanolic enriched extract fractions of increased polarity in mass (g) and their % (w/w) yields.

| Fractions | Mass (g) | Yield (% w/w) |
|---|---|---|
| DCM 100% | 0.0276 | 0.44 |
| DCM / EtOAc (10%) | 0.1636 | 2.64 |
| DCM / EtOAc (50%) | 0.2379 | 3.84 |
| EtOAc 100% | 0.3173 | 5.12 |
| EtOAc / MeOH (10%) **(S10)** | 0.9326 | **15.04** |
| EtOAc / MeOH (20%) **(S20)** | 1.5671 | **25.27** |
| EtOAc / MeOH (50%) **(S50)** | 1.4511 | **23.40** |
| MeOH 100% | 0.2462 | 3.97 |

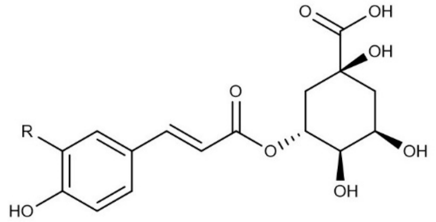

| Phenolic acids | R |
|---|---|
| Feruoylquinic acid (3) | $OCH_3$ |
| Chlorogenic acid (5) | OH |

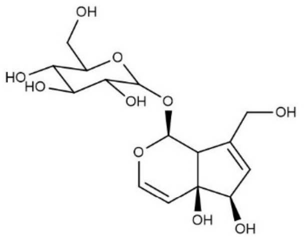

**1,4**

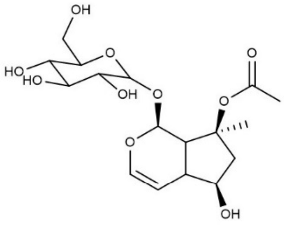

**6**

| Iridoids |
|---|
| Melittoside derivatives (1),(4) |
| Ajugoside (6) |

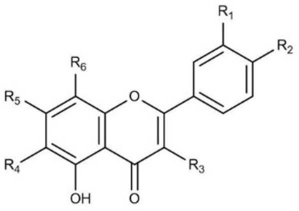

| Flavonoid | $R_1$ | $R_2$ | $R_3$ | $R_4$ | $R_5$ | $R_6$ |
|---|---|---|---|---|---|---|
| Apigenin (17) | H | OH | H | H | OH | H |
| Apigenin-7-*p*-coumaroyl-glucoside (16) | H | OH | H | H | *p*-coumaroyl-glu | H |
| Apigenin-7-*O*-glucoside (11) | H | OH | H | H | *O*-glu | H |
| Isoscutelarein 7-*O*-allosyl-(1→2)-glucoside (12) | H | OH | H | H | *O*-all-glu | OH |
| Luteolin 7-*O*-allosyl-(1→2)-[6''-*O*-acetyl]-glucoside (13) | OH | OH | H | H | *O*-(6''-*O*-acetyl)-β- glu | H |
| 4'-*O*-methylisoscutellarein 7-*O*-allosyl-(1→2)-glucoside (14) | H | $OCH_3$ | H | H | *O*-all-glu | OH |
| 4'-*O*-methylisoscutellarein 7-*O*-allosyl-(1→2) [6''-*O*-acetyl]-glucoside (15) | H | $OCH_3$ | H | H | *O*-(6''-*O*-acetyl)-β- glu | OH |
| 4'-*O*-methylisoscutellarein 7-*O*-[6'''-*O*-acetyl]-allosyl-(1 → 2)-[6''-*O*-acetyl]-glucoside (18) | H | $OCH_3$ | H | H | *O*-[6'''-*O*-acetyl- *β*-D-all-(1→2)] 6''- *O*-acetyl-*β*-*D*-glu] | OH |
| Xanthomicrol or Hypolaetin trimethyl ether (19) | H | OH | H | $OCH_3$ | $OCH_3$ | $OCH_3$ |

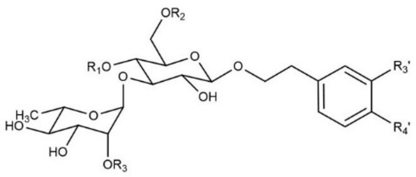

| Phenylethanoid glycosides | $R_1$ | $R_2$ | $R_3$ | $R_3$ | $R_4$ |
|---|---|---|---|---|---|
| Echinacoside (7) | feru | glc | H | OH | OH |
| Lavandulifolioside (8) | caff. | api | api | OH | OH |
| Isoverbascoside (9) | H | caff. | H | OH | OH |
| Leucoseptoside A (10) | feru | H | H | OH | OH |

*feru = feruoyl; caff = caffeoyl; glc = glucose; api = apiose

**Figure S1.** Chemical structures of the identified compounds from S10, S20 and S50 fractions respectively from wild *S. sipylea* Boiss. The numbers in parentheses refer to Table 2 in the main manuscript.

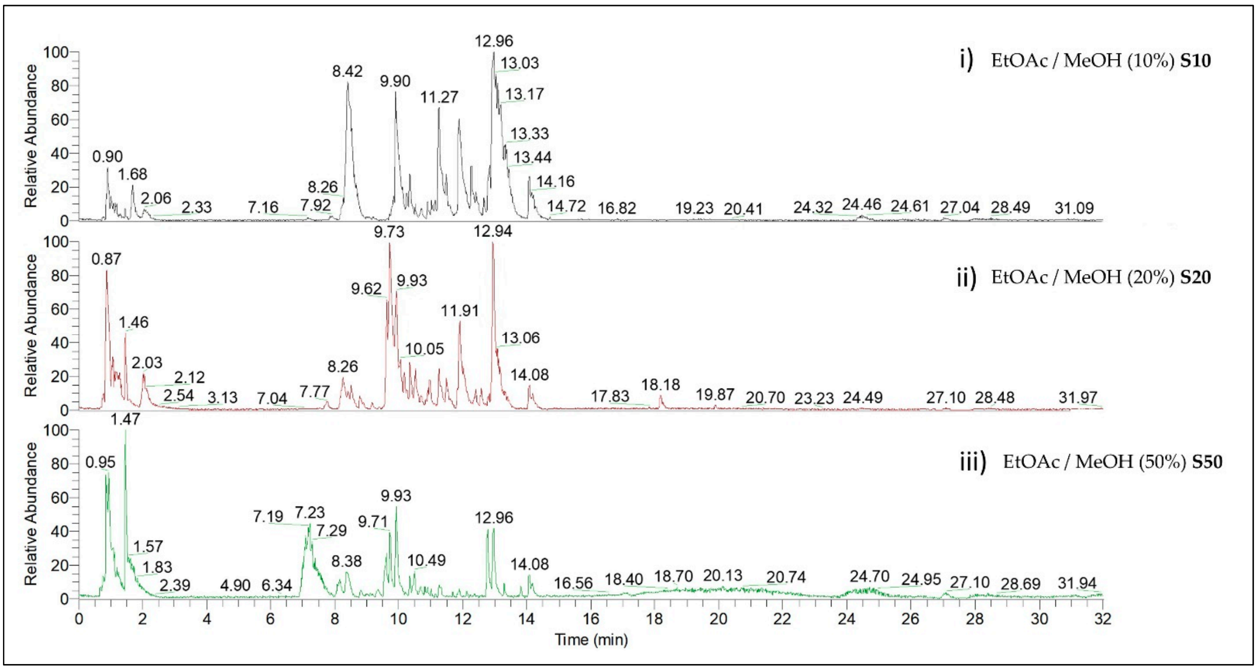


**Figure S2.** UPLC-ESI (-)-HRMS chromatograms of (i) EtOAc / MeOH (10%) (S10), (ii) EtOAc / MeOH (20%) (S20), and (iii) EtOAc / MeOH (50%) (S50) fractions of the methanol extract from wild *S. sipylea*

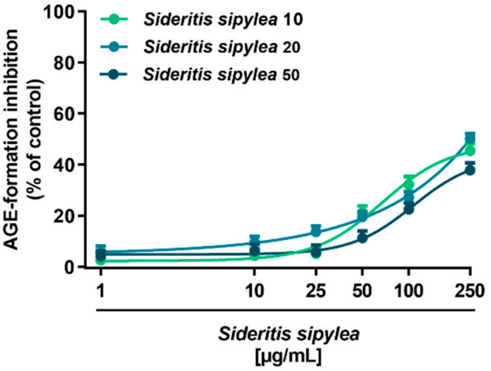


**Figure S3.** AGE formation inhibition by S10, S20 and S50 fractions from *S. sipylea* Boiss. Data represents the average and standard error of at least six replicates from at least two experiments.
